# Insights from the genome of *Ophiocordyceps polyrhachis-furcata* to pathogenicity and host specificity in insect fungi

**DOI:** 10.1186/s12864-015-2101-4

**Published:** 2015-10-28

**Authors:** Duangdao Wichadakul, Noppol Kobmoo, Supawadee Ingsriswang, Sithichoke Tangphatsornruang, Duriya Chantasingh, Janet Jennifer Luangsa-ard, Lily Eurwilaichitr

**Affiliations:** National Center for Genetic Engineering and Biotechnology, National Science and Technology Development Agency, 113 Thailand Science Park, Phahonyothin Rd., Khlong Neung, Khlong Luang, 12120 Pathum Thani Thailand; Department of Computer Engineering, Faculty of Engineering, Chulalongkorn University, Floor 17th, Building 4, Payathai Rd., Wangmai, Pathumwan, 10330 Bangkok Thailand

**Keywords:** *Ophiocordyceps unilateralis*, Genome, Next-generation sequencing, Comparative genomics, Host specificity, Pathogenicity, Pathogen-host interaction

## Abstract

**Background:**

*Ophiocordyceps unilateralis* is an outstanding insect fungus for its biology to manipulate host ants’ behavior and for its extreme host-specificity. Through the sequencing and annotation of *Ophiocordyceps polyrhachis-furcata*, a species in the *O. unilateralis* species complex specific to the ant *Polyrhachis furcata*, comparative analyses on genes involved in pathogenicity and virulence between this fungus and other fungi were undertaken in order to gain insights into its biology and the emergence of host specificity.

**Results:**

*O. polyrhachis-furcata* possesses various genes implicated in pathogenicity and virulence common with other fungi. Overall, this fungus possesses protein-coding genes similar to those found on other insect fungi with available genomic resources (*Beauveria bassiana, Metarhizium robertsii* (formerly classified as *M. anisopliae s.l.*)*, Metarhizium acridum, Cordyceps militaris, Ophiocordyceps sinensis*). Comparative analyses in regard of the host ranges of insect fungi showed a tendency toward contractions of various gene families for narrow host-range species, including cuticle-degrading genes (proteases, carbohydrate esterases) and some families of pathogen-host interaction (PHI) genes. For many families of genes, *O. polyrhachis-furcata* had the least number of genes found; some genes commonly found in other insect fungi are even absent (e.g. Class 1 hydrophobin). However, there are expansions of genes involved in 1) the production of bacterial-like toxins in *O. polyrhachis-furcata*, compared with other entomopathogenic fungi, and 2) retrotransposable elements.

**Conclusions:**

The gain and loss of gene families helps us understand how fungal pathogenicity in insect hosts evolved*.* The loss of various genes involved throughout the pathogenesis for *O. unilateralis* would result in a reduced capacity to exploit larger ranges of hosts and therefore in the different level of host specificity, while the expansions of other gene families suggest an adaptation to particular environments with unexpected strategies like oral toxicity, through the production of bacterial-like toxins, or sophisticated mechanisms underlying pathogenicity through retrotransposons.

**Electronic supplementary material:**

The online version of this article (doi:10.1186/s12864-015-2101-4) contains supplementary material, which is available to authorized users.

## Background

Fungi constitute one of the most diverse kingdoms of living organisms including various forms and ecological roles such as decomposers, mutualists or parasitical symbionts. Their importance for industrial and agricultural applications and their experimental tractability made them useful and popular model for studying cell biology and functional genomics in various biological context. In this regard, the number of whole-genome sequence data from a wide variety of fungi has dramatically increased during the last decade [1].

Entomopathogenic fungi (or insect fungi) are also receiving growing attention with regard to the development of genomic resources. On one hand, *M. anisopliae s.l.* (including *M. robertsii*) and *B. bassiana s.l.*, which are widely used as agents for biological control have instigated the development of genomic resources and experimental approaches in order to study virulence and pathogenicity [2, 3]. On the other hand, other species such as *O. sinensis* and *C. militaris* are widely used in Asia as traditional Chinese medicine and has therefore spurred interests for studying novel biosynthetic pathway and sexual reproduction through genomic approaches [4, 5]. Insect fungi are also well known for the production of interesting secondary metabolites such as polyketides and non-ribosomal peptides whereas the synthetic pathways have not yet been all elucidated [6].

In this study, we report a draft genome of *O. polyrachis-furcata,* a species in the *O. unilateralis* species complex, which is also a Hypocrealean entomopathogenic fungus. This species is outstanding for its ecological strategy as it modifies host ants’ behavior in order to favor its own dispersion (“death grip,” i.e. infected ants climb into vegetation, bite vegetal materials then hang themselves upside down until death, and considered as an extended phenotype from the fungus.) [7]. The biology of *O. unilateralis* still has much to be discovered. The pathogenesis and molecular basis to pathogenicity are poorly known while the molecular basis of behavior manipulation is a total black box. The genome sequencing of this species will provide a basis for further exploration on these issues.

Additionally, this fungus also differs from those previously cited regarding its host range. While *M. anisopliae* (including *M. robertsii*) and *B. bassiana* have very broad host ranges infecting several insect orders and could live as plant endophytes and in the soil, *O. sinensis*, *C. militaris* and *O. unilateralis* have narrower host ranges including various lepidopteran families for *C. militaris* [8], a family of Lepidoptera (Hepialidae) for *O. sinensis* [9], and only a tribe (Camponotini) in a sub-family of ants (Formicinae) for *O. unilateralis* [10]. Previous studies showed hidden diversity of *O. unilateralis* associated to ant species, suggesting a diversification through host specificity [10, 11]. A recent study suggested that *O. unilateralis* in Thailand should be subdivided into three distinct species (*O. polyrhachis-furcata*, *O. camponoti-saundersi*, *O. camponoti-leonardi*) according to the host species [11]. In this study, we focused on sequencing *O. polyrhachis-furcata* and analyzing its genes that were previously reported to be important to different steps of pathogenesis and thus virulence in other insect fungi and fungal pathogens. Through comparative genomics between our fungus and nineteen other fungi including other entomopathogenic fungi (*B. bassiana*, *M. robertsii*, *M. acridum*, *C. militaris*, *O. sinensis*), some fungal plant pathogens (*Magnaporthe oryzae*, *Botryotinia fuckeliana*, *Fusarium graminearum*, *Sclerotinia sclerotiorum*, *Ustilago maydis*, *Verticillium alfalfae*), opportunistic human pathogenic fungi and yeast (*Aspergillus fumigatus*, *Candida albicans*, *Saccharomyces cerevisiae*), a fungal pathogen of amphibian (*Batrachochytrium dendrobatidis*) and saprophytic fungi (*Aspergillus nidulans*, *Neurospora crassa*, *Coprinopsis cinerea*) as well as a non-pathogenic yeast (*Schizosaccharomyces pombe*); we identified genes involved in various steps of pathogenesis, investigated the common attributes of being entomopathogenic and the extent to which the host ranges have shaped their evolution as well as enable the discovery of new biosynthetic pathways.

## Results

### General genome features

The genome of *O. polyrhachis-furcata* (strain BCC54312) was sequenced in-house using the Roche 454 GS FLX system. This resulted in 1.28 Gb of sequence data (37x coverage) with average read length of 652 bp. The shotgun reads were de novo assembled resulting in 4419 contigs. For sequence scaffolding, a DNA library of 3 kb, 6 kb, and 8 kb inserts were constructed and sent to Macrogen (Seoul, Republic of Korea) for sequencing on Hiseq2000 platform (Illumina). 4419 contigs together with the mate-pair libraries were assembled into 418 scaffolds (59 scaffolds > 1 kb; N50 3.3 Mb) with a total estimated genome size of about 43 Mb. The assembled genome has been deposited as an NCBI’s Whole Genome Shotgun (WGS) project under accession number LKCN00000000 and the data of the sequenced samples deposited at the NCBI’s BioSample database under the accession number SAMN04099149.

While having an equivalent genome size to other insect fungi (except *O. sinensis*), the genome of *O. polyrhachis-fucata* BCC54312 was predicted to have 6793 protein coding genes. This number is close to that of protein coding genes reported in [4] for *O. sinensis* and substantially less than those reported for *M. robertsii*, *M. acridum*, *B. bassiana*, and *C. militaris* which have broader host ranges. Furthermore, the number of secreted proteins as predicted by SignalP 4.0 [12] is less than half of those four entomopathogenic genomes (Table 1)*.* This number is reliable as the assembled genome was assessed to be as complete as 96 % (4.5 % duplicated), 2.5 % fragmented, and only 1.1 % missing. The set of annotated protein coding genes was assessed to be 95 % complete (9.2 % duplicated), 2.8 % fragmented, and only 1.8 % missing.

**Table 1:** Comparison of genome features among six entomopathogenic fungi

Features	OPF	OPSINE	METANI	METACR	BEUBAS	CORMIL
Size (Mb)	43	~120	39	38.1	33.7	32.2
Coverage (fold)	37x(454)	241x	100x	107x	76.6x	147x
No.of scaffolds(>1 kb)	65	-	176	241	242	13
Scaffold N50 (Mb)	3.3	-	~2.0	0.33	0.73	4.55
%G + C content	45.2	46.1	51.5	50.0	51.5	51.4
%G + C in coding genes	59.7	-	54.4	54.1	56.6	58.6
Protein coding genes	6,799	6,972	10,582	9,849	10,366	9,684
Exons per gene	3.29	2.6	2.8	2.7	2.7	3
tRNAs	83/77	-	141	122	113	136
No. of secreted proteins	690	-	1,865	1,490	~1886	1,572
No. of PHI genes	1,223	998	1,828	1,629	-	1,547
No. of Pth11-like GPCRs	14	-	54	40	23	18

**Fig. 1:**
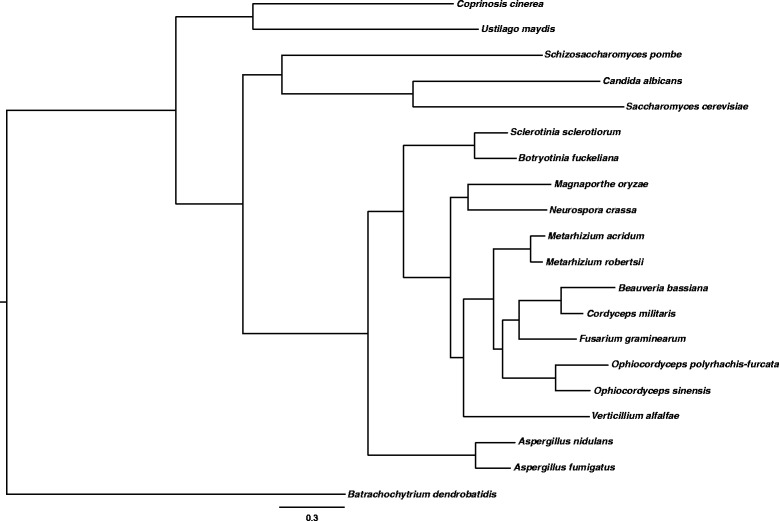
A phylogenomic tree based on conserved proteins among taxa

Table 1 shows the overall genome features of *O. polyrhachis-furcata* compared with other five entomopathogenic fungi using the same versions of computer programs and databases except for protein secretion. A phylogenomic tree based on conserved proteins among all taxa used in this study showed *O. polyrhachis-furcata* to be closely related to *O. sinensis* which is from the same family (Ophiocordycipitaceae) and clustered with other fungi from the order Hypocreales including other entomopathogenic fungi (Fig. 1).

### Pathogen-Host Interaction genes

**Fig. 2:**
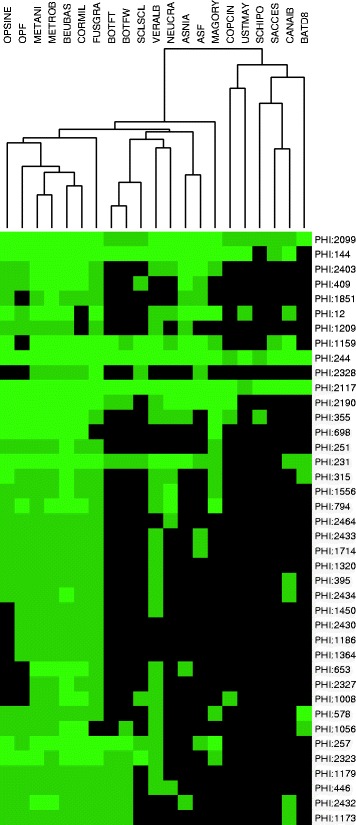
A phylogram based on the number of genes in all PHI families with presence/absence of some selected families. OPF = *Ophiocordyceps polyrhachis-furcata*, OPSINE = *Ophiocordyceps sinensis*, BEUBAS = *Beauveria bassiana*, CORMIL = *Cordyceps militaris*, METANI = *Metarhizium anisopliae*, METROB = *Metarhizium robertsii*, FUSGRA = *Fusarium graminearum*, VERALB = *Verticillium alfalfae*, SCLSCL = *Sclerotinia sclerotiorum*, BOTFW = *Botryotinia fuckeliana*, ASNIA = *Aspergillus nidulans*, ASF = *Aspergillus fumigatus*, SACCES = *Saccharomyces cerevisiae*, CANAlB = *Candida albicans*, SCHIPO = *Schizosaccharomyces pombe*, COPCIN = *Coprinosis cinerea*, USTMAY = *Ustilago maydis*, BATD = *Batrachochytrium dendrobatidis*

The potential virulence-associated genes in the *O. polyrhachis-furcata* and nineteen other genomes were identified by BLAST analysis against the pathogen-host interaction (PHI) database [13]. This is a useful database synthesizing a plethora of experimentally verified genes related to the mediation of fungal pathogens, including Oomycetes, to cause disease and to provoke response from the hosts (pathogenicity, virulence and effector genes). We identified 1890 putative PHI genes in *O. polyrhachis-furcata*, which is remarkably lower than in other insect fungi with broader host ranges (see Additional file 1: Table S1). The six species of entomopathogenic fungi were clustered together according to the number of specific PHI genes, shown to be related to pathogenicity, which were identified across all the genomes used for the analysis (Fig. 2). Particularly, *O. polyrhachis-furcata* was the closest to *O. sinensis*. Also, the plant pathogens appeared close in terms of PHI genes abundance while yeasts (*S. cerevisiae, S. pombe, C. albicans*), saprophytic mushroom (*C. cinerea*) and the amphibian pathogen (*B. dendrobatidis*) share similar patterns of PHI genes abundance (Fig. 2 and Additional file 1: Table S1).

Although *O. polyrhachis-furcata* and *O. sinensis* have generally less PHI genes compared with the other insect fungi, some PHI gene families were specifically found or in higher numbers in these two fungi. Interestingly, *O. polyrhachis-furcata* contains more PHI:871 genes (8), compared with other five entomopathogenic fungi (0 to 1). This class of genes was reported as virulence gene in the rice blast fungus, *M. grisea* [14]. Among the twenty genomes, all except *O. polyrhachis-furcata* and *M. grisea*, contain at most two PHI:871 genes. Among the six entomopathogenic fungi, PHI:113, PHI:1158, PHI:1276, PHI:2314, PHI:449 and PHI:1278 genes were found only in *O. polyrhachis-furcata*. PHI:113 was also reported in *M. grisea*. Its disruption caused the loss of pathogenicity against rice [15]. PHI:1158 was characterized in *Mycosphaerella graminicola* as resistance to azole fungicides [16]. The gene deletion of PHI:2314 resulted in reduced virulence of *S. sclerotiorum* and oxidative burst in adjacent uninfected cells of tomato (*Nicotiana benthamiana*) [17]. The disruption of PHI:449 gene in *Claviceps purpurea T5* reduced virulence against Rye [18] while the disruption of PHI:1276, PHI:1278 in *Gibberella zeae* did not affect pathogenicity [19]. *O. polyrhachis-furcata* and *O. sinensis* contain PHI:292 [20], PHI:2198 [21], PHI:2222 [22], PHI:2342 [23] and PHI:2534 [24] genes, which are missing from the other four entomopathogenic fungi. The deletion of all of these genes except PHI:2342 resulted in reduced-virulence phenotypes in other fungal pathogens. For example, the lethality effect on *A. fumigatus Af293* comes from the double mutant of *PHI:2534* (the deletion of both *ERG11A* and ERG11B) while each individual deletion can compensate the loss of the other [24].

*B. bassiana* contain more copies of PHI:1139 (79), PHI:820(23), PHI:821(19), PHI:823 (10), compared to other five entomopathogenic fungi (5–15 for PHI:1139 and 1–2 for PHI:820, PHI:821, and PHI:823). Interestingly, PHI:1139 in *Xanthomonas oryzae* is a plant avirulence determinant [25]. The mutant phenotype of other three PHI genes is resistant to chemical fungicides [26–28].

Another interesting PHI genes set consists of PHI:2240, PHI:1555, PHI:812, and PHI:511 as *O. polyrhachis-furcata* and *O. sinensis* have less number of these PHI genes compared to other four entomopathogenic fungi. PHI:2240 was reported as a plasma membrane-localized sucrose transporter (Srt1); a fungal virulence factor, characterized from corn smut fungus *U. maydis* [29]. PHI:1555 has not been reported to affect pathogenicity while PHI:812 was reported as a virulence gene in *M. grisea* [14]. Finally, PHI:511 was reported as involving in drug sensitivity and virulence in *C. albicans* [30] (see Additional file 2: Table S2).

### Surface adhesion genes

The first step to cause disease is the attachment of the infective propagules on the hosts’surface. This involves hydrophobic interactions between the spore surface proteins (e.g. adhesin, hydrophobin) and the lipid layer on the epicuticle of insects. Hydrophobins can be classified into two classes based on their hydropathy and solubility characteristics [31]. In insect fungi, hydrophobins were previously shown to be involved in the formation of appressorium in *M. anisopliae* [32] and *B. bassiana* [33, 34] as well as virulence factors in *M. brunneum* [35]. Only Class 2 hydrophobins, which are represented by the orthologous proteins MAA_01182 and MAC_09507 in *M. robertsii* and *M. acridum* respectively, were identified in the genome of *O. polyrhachis-furcata*. The genomes of the three other entomopathogenic fungi also contain this class of hydrophobins. In addition, six other species (*B. fuckeliana*, *F. graminearum*, *M. oryzae*, *N. crassa*, *S. sclerotiorum* and *V. alfalfae*) have orthologous proteins of this class. Notably, the protein BBA_03071 of *B. bassiana*, which is annotated as Hydrophobin-like protein at UniProt, was clustered to the same orthologous group of the Class 2 hydrophobins of the other entomopathogenic fungi while the annotated Hydrophobin 2 of this species (BBA_00530: *hyd2*) could not be clustered with the other species. The gene product of *hyd2* was reported as the major component of the *B. bassiana* rodlet layer [33]. In contrast, no orthologous Class 1 hydrophobin was identified in *O. polyrhachis-furcata*. The Class 1 hydrophobins MAA_10298 and MAC_04376, reported in [2], and BBA_03015, reported in [3], were identified in *C. militaris* and *O. sinensis* as well as in eight other species, including *A. fumigatus*, *A. nidulans, B. fuckeliana* (strain BcDW1), *C. cinerea* (strain Okayama-7/130/ATCC MYA-4618/FGSC 9003), *Gibberella zeae*/*F. graminearum* (anamorph), *M. oryzae*, *S. sclerotiorum*, *U. maydis*.

Beside hydrophobins, the MAD1 adhesin (MAA_03775), characterized previously in *M. robertsii*, also provides adhesion specifically to insect host surfaces, enhances the expression of genes related to i.e., germination, blastospore formation, and thus affects virulence to caterpillars [36]. *O. polyrhachis-furcata* as well as all other eighteen species have multiple orthologous proteins of this adhesin. The MAD2 adhesin (MAA_03807) allows adhesion of *M. robertsii* to plant surfaces [36] but has no effects on fungal differentiation and entomopathogenicity. Interestingly, only six genomes, the *V. alfalfae* genome together with five other from entomopathogenic fungi, excluding *O. sinensis* have a single copy of MAD2 adhesin ortholog.

### Insect cuticle degrading genes

**Fig. 3:**
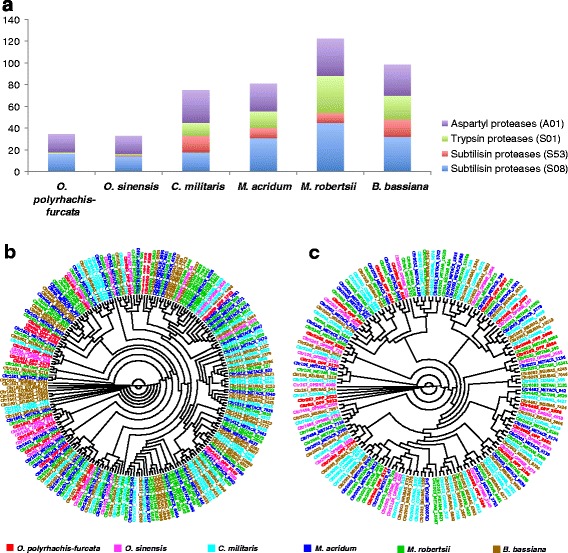
Expansions of proteases among six entomopathogenic fungi including *O. polyrhachis-furcata*. **a** The distribution of proteases families among the six entomopathogenic fungi included in this study. **b** A maximum likelihood-based unrooted phylogenetic tree illustrating the relation between orthologous proteins of subtilisins (S08 and S53). **c** A maximum likelihood-based unrooted phylogenetic tree illustrating the relation between orthologous proteins of aspatyl proteases (A01). The sequences labels; colored following insect fungi species: red = *O. polyrhachis-furcata* (OPF), brown = *B. bassiana* (BEUBAS), green = *M. robertsii* (METANI), dark blue = *M. acridum* (METACR), light blue = *C. militaris* (CORMIL), pink = *O. sinensis* (OPSINE); are written in the form of cluster id._fungal species_sequences id. The cluster identities were obtained inferred according to Inparanoid/QuickParanoid [38, 39]

Insect pathogens are expected to secrete large number of enzymes for degrading insect cuticle. These include various proteinases, particularly subtilisins, and other enzymes susceptible to degrade molecules present on the insects’ cuticles (i.e. chitinase and other glycoside hydrolases). *O. polyrhachis-furcata* and *O. sinensis* contain similar numbers of subtilisins, trypsins, and aspartyl proteases which are much smaller than those of other four entomopathogenic fungi (Additional file 3: Table S3 and Fig. 3a). In *M. anisopliae s.l.,* subtilisins were reported to degrade host cuticles and allow acquiring nutrients, with potential functional differences between subfamilies in secondary substrate specificities and adsorption properties [37]. *O. polyrhachis-furcata*, *O. sinensis* and *C. militaris* contain about half the number of subtilisins (S08) compared with *B. bassiana*, *M. robertsii*, and *M. acridum*. Eleven out of twenty-three subfamilies of subtilisins, were not identified in any of the six entomopathogenic species while four are common across all six species (Additional file 4: Table S4). Among those that were commonly found in the six insect fungi, subfamily S08.UPA (subfamily S8A unassigned peptidases) is very abundant.

Regarding the subtilisins S53 (sedolisins), four out of five subfamilies could be identified in entomopathogenic fungi. *O. polyrhachis-furcata* and *O. sinensis* contain only one protease in the subfamily S53.UPW (family S53 unassigned peptidases), which is common across the six entomopathogenic fungi. Four other entomopathogenic species contain a few more proteinases, including aorsin (S53.007) and grifolisin (S53.010) with one additional scytalidolisin (S53.011) for *C. militaris*, which were not found in *O. polyrhachis-furcata* and *O. sinensis*. The subfamily S53.003 (tripeptidyl-peptidase I) was not identified in any six species.

The subtilisins (S08 and S53) of *O. polyrhachis-furcata* derived from those of other entomopathogenic fungi do not form a monophyletic clade (Fig. 3a), indicating that they have evolved independently from the subtilisins of other entomopathagenic fungi. Five of them (OPF_4096, OPF_2781, OPF_4408, OPF_1157, OPF_4650) could not be assigned to any orthologous group with any other fungi. These proteins were respectively annotated as S08.053 (MER006091:oryzin) (*Aspergillus flavus*), S08.UPA (MER032872: subfamily S8A unassigned peptidases) (*M. grisea*)*,* S08.053 (MER087525:oryzin) (*Aspergillus clavatus*), S08.056 (MER126013:cuticle degrading peptidase of parasitic fungus) (*Hirsutella minnesotensis*), and S08.056 (MER123475:cuticle degrading peptidase of parasitic fungus) (*O. sinensis*). However, it is to be noted that orthologous groups of subtilisins inferred by Inparanoid/QuickParanoids [38, 39] are not supported by the maximum likelihood-based phylogenetic inference (Fig. 3), suggesting that the similarity-based method of Inparanoid/QuickParanoid should be taken with caution.

Both *O. polyrhachis-furcata* and *O. sinensis* contain only one trypsin S01.UPB (subfamily S1B unassigned peptidases), which is conserved across six entomopathogenic fungi. Other insect fungi species have substantially higher number of trypsins (22 in *B. bassiana*, 34 in *M. robertsii*, 15 in *M. acridum* and 12 in *C. militaris*). Subfamily S01.UPA (subfamily S1A unassigned peptidases) was identified in all but the two *Ophiocordyceps* and the most abundant family. Subfamily S01.412 (CHY1 peptidases or chymotrypsins) was identified in *B. bassiana* (BBA_02727), *C. militaris* (CCM_08282) and *M. robertsii* (MAA_07484) but not in *M. acridum.* Additionally, only *B. bassiana* contains additional two Nma111 peptidases (S01.434).

The numbers of aspartyl proteases (A01) identified in *O. polyrhachis-furcata* and *O. sinensis* are about half of the other four entomopathogenic fungi and in only four out of thirty-five subfamilies. Subfamily A01.UPA (subfamily A1A unassigned peptidases) is conserved across the six species and very abundant. Twenty-two out of thirty-five subfamilies were not identified in any six species. Subfamilies A01.018 (saccharopepsin) and A01.077 (CtsD peptidase) were found in all but the two *Ophiocordyceps* species. Several subfamilies A01.017 (endothiapepsin), A01.027 (trichodermapepsin), A01.057 (MernameAA034 peptidase), A01.079 (PepAa peptidase), and A01.UPB (subfamily A1B unassigned peptidases) were identified in only the two *Metarhizium* species. Additionally, A01.082 (SA76 peptidase) was identified in all except *O. sinensis* (Additional file 4: Table S4). Like subtilisins S08 and S53, the aspatryl proteases of *O. polyrhachis-furcata* do not form a monophyletic clade (Fig. 3b).

Like proteases, chitinases have been reported as involved in host penetration which is a crucial first step of pathogenesis and to influence the virulence [40–42]. The chitinases are classified into a family of Glycoside Hydrolase (GH18). GH18 is the most abundant family of Glycoside Hydrolase found in all fungi included in our analysis (Additional file 5: Table S5). Among entomopathogenic fungi, broad host-range species including *B. bassiana* and *M. robertsii* also possess more genes encoding this GH family (195 and 179 respectively) than *M. acridum* (160), *C. militaris* (157), *O. sinensis* (110) and also *O. polyrhachis-furcata* (134). Additionnally, *O. polyrhachis-furcata* and *O. sinensis* have less proteins of several GH families than the other four entomopathogenic fungi (GH16, GH3, GH4, GH76, GH92, GH43, GH78, GH79: Additional file 5: Table S5). Some GH, and Polysaccharide Lyase families were reported to be related to host-specific adaptation in plant pathogens (i.e. GH6, GH7, CBM1, GH28, GH78, GH88, GH95, PL1, PL3 [43]). All six entomopathogenic fungi do not have the GH7 families, less than half of the numbers of GH28, GH78, GH95 (Additional file 5: Table S5), and many less or no PL1 and PL3 (Additional file 6: Table S6). Among these families; GH28, GH78 showed the same tendency as that found for GH18 in that broad host-range species possess more genes than narrow host-range species including *O. polyrhachis-furcata*. Regarding the families of carbohydrate esterases (CE), on one hand, *O. polyrhachis-furcata* and *O. sinensis* have less number of CE for the families CE1 and CE6, compared to the other four entomopathogenic fungi species. On the other hand, they contain similar number of proteins to the other insect fungi but different to plant pathogens for the CE3, CE5 and CE12 families (Additional file 7: Table S7).

### Bacterial-like toxins

Entomopathogenic fungi are expected to infect hosts through cuticular penetration and thus to possess less toxins compared with other pathogens such as bacteria or viruses. However, the number of bacterial-like toxin proteins and toxin-biosynthesis proteins found in *O. polyrhachis-furcata* are relatively high (22), compared to *C. militaris* (9), *M. robertsii* (34), *M. acridum* (10) and *O. sinensis* (9); this number is actually the same as *B. bassiana* [3] (Additional file 8: Table S8). *M. robertsii* has the highest number of such proteins (34). Seventeen out of twenty-two toxins found in the genome of *O. polyrhachis-furcata* are heat-labile enterotoxins A chain. Others include a zeta toxin, a ribotoxin, two cholera entorotoxins and a killer toxin. Among these toxins, a gene putatively encoding a heat-labile A chain was identified in front of a NRPS gene in a predicted NRPS gene cluster. The prevalence of genes coding for bacterial-like toxins in the genome of *O. polyrhachis-furcata* suggests the possibility of oral toxicity as a mode of killing insect host.

### Signal transduction

The encounter between the pathogens and the hosts will inevitably engage the signal transduction from both parties. We also found in the genome of *O. polyrhachis-furcata* various genes related to cellular signal transduction including G proteins, G protein-coupled receptors (GPCRs) and histidine kinases (HK).

Among six entomopathogenic fungi, *O. polyrhachis-furcata* contains the smallest number of Pth11-like G-protein coupled receptors (GPCRs) which are cell-surface integral membrane proteins required for pathogenicity [44]. Also, as PHI genes (PHI-base accession: PHI:404 and PHI:441), they mediate cell response to inductive cues [45]. PHI genes were shown to be related to host specificity of *M. acridum* via differential expression on locust and cockroach cuticles [2]. All other fourteen non-entomopathogenic fungi clearly contain less number of Pth11-like genes.

Two PHI:441 proteins in *O. polyrhachis-furcata* out of seven, OPF_8495 (PHI:441|BTP1|CAE55153|TX:40559|Botrytis cinerea) and OPF_6721 (PHI:441|BTP1|CAE55153|TX:40559|Botrytis cinerea), have no orthologs with any other nineteen compared species. Based on BLASTX against NR database, the two proteins were annotated as “hypothetical protein THITE_2110904” and “putative integral membrane protein [Eutypalata UCREL1]”. Meanwhile, six putative PHI:404 genes were found in *O. polyrhachis-furcata* and could be clustered into orthologous groups with the other fungi.

G protein alpha subunits have been comprehensively studied, as a key component of signal transduction pathways and pathogenicity [46]. Its disruption in *Stagonospora nodorum* made the fungus less pathogenic, unable to sporulate, and albino phenotype with secretion of brown pigments into growth media [47]. In *Metarhizium*, G-alpha proteins in *M. robertsii* (MAA_03488) and *M. acridum* (MAC_04984) were observed as the most expressed G-alpha genes during their infection of either cockroach or locust cuticles [2]. These two genes are clustered together as orthologous proteins with all remaining eighteen species including *O. polyrhachis-furcata*: OPF_3427. The two-component Histidine Kinase (HK) signaling pathways mediate environmental stress responses and regulation of secondary metabolism [48, 49], response to bacterial metabolites [50], hyphal development [51], virulence [52] and sensitivity to dicarboximide and phenylpyrrole fungicides [53, 54] in diverse fungal species. *O. polyrhachis-furcata* contains the similar number of histidine kinases (9) compared with *M. robertsii* (10) and *M. acridum* (9) reported in [2].

### Core genes involved in the biosynthesis of secondary metabolites

Insect fungi are also well known for producing a variety of secondary metabolites. Genes implicated in their biosynthesis have received large attention, particularly polyketides and non-ribosomal peptides synthetases (PKS and NRPS) [6]. Based on SMURF [55], the *O. polyrhachis-furcata* genome encodes 4 putative NRPS, other 4 NRPS-like, 14 PKS, 1 PKS-like, 1 NRPS-PKS hybrid and without dimethylallyl tryptophan synthase (DMAT) genes (Table 2). The total number of 24 core genes in this species is much less than that of *M. robertsii* (60) and *M. acridum* (42) [2] and somewhat less than that of *B. bassiana* (36) [3] and also *C. militaris* (28) [5]. Similar to *M. robertsii*, one of the four putative NRPS-like genes in *O. polyrhachis-furcata* is an antibiotic synthetase, reported for preserving the cadaver from microbial competitors [2]. Another NRPS-like protein (OPF_1123) is mostly similar to another antibiotics synthase (Linear gramicidin synthase subunit D of *F. oxysporum*). The unique PKS/NRPS hybrid gene gets the top hit to fusarin C cluster-polyketide synthase/NRPS, which is a mycotoxin produced by several *Fusarium* species with carcinogenic effects [56]. Beside these core genes, three additional genes (fus2, fus8, and fus9), reported to be responsible for fusarin production [57], were also identified within this hybrid cluster. *O. polyrhachis-furcata* possesses an NRPS (OPF_4495), which is similar (39 % identity) to peramine synthetase (PerA) of *Epichloe festucae*, the essential enzyme for the biosynthesis of peramine; a compelling insect feeding deterrent to protect their grass host from insect herbivory [58]. Based on antiSMASH [59], *O. polyrhachis-furcata* also possesses the PKS-like and NRPS-like which are respectively similar to Ochratoxin A polyketide synthase (35 % identity) and a non-ribosomal peptide synthetase (36 % identity) of *Penicillium nordicum* characterized in [60]. Even located on the same scaffold, they are not on the same cluster.

**Table 2:** Number of core genes involved in the biosynthesis of secondary metabolites

Fungi	DMAT	HYBRID	NRPS	NRPS-like	PKS	PKS-like	Total
*O. polyrhachis-furcata*	0	1	4	4	14	1	24
*C. militaris*	1	3	5	8	9	2	28
*M. acridum*	3	1	13	8	13	4	42
*M. robertsii*	5	5	14	9	24	3	60
*B. bassiana*	0	3	13	7	12	1	36
*B. cinerea*	1	0	6	8	16	6	37
*S. sclerotiorum*	1	0	5	5	16	2	29
*F. graminearum*	0	1	10	11	14	1	37
*E. festucae*	4	1	18	9	11	3	46
*N. crassa*	1	0	3	3	6	2	15
*M. oryzae*	3	3	5	6	12	3	32
*A. nidulans*	6	1	11	12	24	4	58
*A. fumigatus*	7	1	13	5	13	1	40

This table is extended and rearranged from Table S13 in [2] by adding *O. polyrhachis-furcata*’s identified core genes following SMURF [55]. Core genes of *C. militaris* and *B. bassiana* were excerpted from Table 3 in [5] and Table 2 in [3], respectively. DMAT: Dimethylallyl tryptophan synthase, NRPS: non-ribosomal peptide synthetase, HYBRID, hybrid PKS–NRPS enzyme

### Repeat elements and transposases

**Fig. 4:**
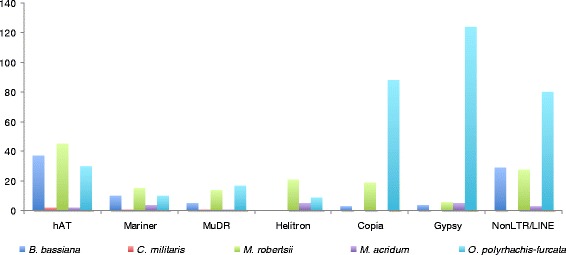
The numbers of transposases between the genomes of different entomopathogenic fungi. The data of *O. sinensis* [4] were not included in this graphic as this species contain much higher numbers of most transposable elements classes (Additional file 9: Table S9) and skewed the representation

On one hand, the genome of *O. polyrhachis-furcata* comprises notably more Class II transposable elements (TEs) - the DNA transposons including DNA/hAT (23), DNA/Mariner (7), DNA/MuDR (11), and DNA/Helitron (7) - compared with the genomes of *C. militaris* and *M. acridum* which contain less than five of these elements in their genomes. On the other hand, *O. polyrhachis-furcata* possess less of these elements compared to the genomes of *B. bassiana* and *M. robertsii* (Fig. 4 and see Additional file 9: Table S9) except for DNA/Helitron of which *B. bassiana* is devoid. Particularly, *M. robertsii* contains several more DNA/Helitron (26) compared with *O. polyrhachis-furcata* (9) and *M. acridum* (5) while *C. militaris* do not have this specific element. Regarding the Class I elements or the retroelements, the LTR/Copia, LTR/Gypsy, as well as the Non-LTR/LINE are much more abundant in *O. polyrhachis-furcata* compared to those species. Nevertheless, except for DNA/hAT and DNA/helitron, *O. sinensis* contain many more transposable elements than the other insect fungi species studied here (Additional file 9: Table S9). Transposases were reported to be expressed during infection in *Metarhizium* [2]. Therefore, this type of genomic mobile element may be related to pathogenicity.

## Discussion

We present here a genome draft of an outstanding insect fungus, *O. unilateralis s.l.* from the host *Polyrhachis furcata*. In our study, we compare this genome to other available fungal genomes of various origins in order to gain insights into the biology of this species. The particularity of this fungus is its extreme host-specificity as shown by the fact that different species are associated to different ant species [10, 11]. Here, we have sequenced *O. polyrhachis-furcata* which is specific to an ant species while, for the others, the degree of host range is variable, ranging from families of the same insect orders (e.g. *O. sinensis* and *O. militaris*) to several insect orders (e.g. *B. bassiana*, *M. robertsii*).

We found that the genome of *O. polyrhachis-furcata* contains various genes which were shown to be implicated during the pathogenesis of fungal pathogens. Many of them are in common with other entomopathogenic fungi and fungal plant pathogens.

In this genome, we observed contractions of several kinds of genes, compared to broad host range entomopathogenic fungi. This signature of genes contraction is found for genes involved throughout the pathogenesis, from genes required for the adhesion of infective propagules to the synthesis of metabolites potentially necessary for coping with insect immune systems. This may have critical implications in the life of this fungus. For example, hydrophobins Classes 1 and 2 are amphiphilic proteins, mostly involved in the mediation of the contact between the fungal cells and hydrophobic/hydrophilic surfaces. Although the roles of both classes and different paralogs are not yet totally elucidated and partially overlapping [31], the disruption of a number of hydrophobins resulted in altered hydrophobicity of the mycelia as well as the morphology and production of conidia [33–35]. The absence of hydrophobin Class 1 in *O. polyrhachis-furcata* could be related to fastidious growth and sporulation of this species in standard complex media while *B. bassiana*, *M. spp.*, *C. militaris* and *O. sinensis* are all fast growers and can produce spores abundantly in the laboratory.

Comparison with extensive available fungal genomes allowed us to grasp common aspects and differences between plant and insect pathogens. A notable difference is the absence of MAD2 adhesin in plant pathogens, which is not the case for the entomopathogenic fungi except *O. sinensis*. This suggests that the entomopathogenic fungi may have dual ways of life, being parasitic to insects and in the same time having the capacity to be endophytes in plants. This has already been shown in *B. bassiana* and *M. anisopliae* [61, 62]. The absence of MAD2 in plant pathogens suggests a totally different mechanism of adhesion. Furthermore, notable differences in numbers of genes are found for various Glycoside Hydrolase (GH), Carbohydrate Esterase (CE) families as well as Polysaccharide Lyase (PL) families. Overall, the comparative analysis shows a cleavage between insect and plant pathogens in the penetration of host’s cell. This may be linked to the fact that fungal pathogens of plants need to overcome the cell wall which has different biochemical composition to insect cuticle.

Subtilisin proteases have been shown to play crucial role in regulating the specificity to hosts of entomopathogenic fungi via the reduction of isoforms [37] or the differential expressions of isoforms according to substrates [63]. Hosts play important role in the evolution of subtilisin-like proteases in entomopathogenic fungi by imposing constraints into the realized niches *in vivo*. In *Metarhizium*, generalist strains were shown to have more paralogous isoforms than a specialist strain [37]. According to our comparative analysis, there is also a tendency in the reduction of subtilisins proteases genes for narrow-host range species of insect fungi with *O. polyrhachis-furcata* having in effect the narrowest host range and one of the lowest numbers of genes coding for subtilisins proteases compared with the other entomopathogenic fungi. This is in accordance with the pattern of the reduction in genes number, found in GH and CE as well as in other genes coding for cuticle-degrading proteolytic enzymes like trypsin and aspatyl proteases, for narrow-host range species. This suggests a direct association between host range and diversity of cuticle-degrading enzymes. Altogether, this consistently supports the early host recognition events as key to host specificity and the tendency toward reduced genes numbers associated with narrow host range.

Referred to the PHI genes classification, we observed also that *O. polyrhachis-furcata* and *O. sinensis* had substantially less genes for some families when compared to the other entomopathogenic fungi. One of these families is clearly related to the membrane transport of sucrose (PHI:2240). The difference in number of genes for this family may result from the affinity to different nature of carbon sources in different insect hosts. Another family of PHI genes (PHI:511), which has different numbers of genes between *O. polyrhachis-furcata*/*O. sinensis* and the broad host range species, is involved in drug sensitivity. Systemic immune response in different hosts may influence on the evolution of this family of genes in entomopathogenic fungi. Other families of PHI genes showing substantial reduction of gene numbers in *O. polyrhachis-furcata* are PHI:404 and PHI:441 which are also Pth11-like G-protein coupled receptors (GPCRs) previously shown to be related to host specificity. *O. polyrhachis-furcata* has the lowest number of Pth11-like genes compared to other five entomopathogenic fungi.

The genes contraction in these various families suggest that specific species like *O. polyrhachis-furcata* may have lost a plethora of proteins of these particular families which potentially cope with diverse systemic immune responses from unspecific host.

An unexpectedly high number of genes related to biosynthesis of bacterial-like toxins are found in the genome of *O. polyrhachis-furcata*, compared to other entomopathogenic fungi species considered as having narrow host range (*M. acridum*, *C. militaris*). Previously, comparative genomics between generalist and specialist strains in *M. anisopliae s.l.* (subsequently *M. robertsii* and *M. acridum*) [64] indicated that specialists had reductive evolution of genes involved in toxin biosynthesis, suggesting that they had lost the capacity to kill host rapidly and to exploit host saprotrophically. Instead, they got specialized into strategies optimizing the struggle against host immune system and exploitation of living hosts. Furthermore, our comparative analysis also supports this hypothesis regarding *M. robertsii* and *M. acridum* which differ in their host ranges and diversity of putative genes producing toxins. Therefore, we expect *O. polyrhachis -furcata* which is an extreme specialist to have evolved into the latter strategy while the data support for the former. This suggests that *O. unilateralis* may have a totally different mechanism for killing host from that of *M. anisopliae*. Toxins found in *O. polyrhachis-furcata*’s genome are for many enterotoxins, suggesting that oral toxicity may constitute a mode of killing for our fungus. However, how *O. polyrhachis-furcata*, as a species of *O. unilateralis* which is well documented for its extended phenotypes in manipulating the ant host, strives to compromise between rapid killing by toxin and slow killing enabling the host manipulation remains to be solved.

Another distinctive feature of *O. polyrhachis-furcata*’s genome is the composition of transposable elements. The LTR/Copia and LTR/Gypsy are much more abundant in *O. polyrhachis-furcata* than in the other insect fungi except *O. sinensis* which has the highest numbers of transposable elements of almost all classes. It is well known that transposable elements are important source of genetic variability of different species and also contribute to intra-specific diversification in various fungi [65]. Also, transposable elements were shown to have important roles in pathogenicity and virulence for many microorganisms. Particularly, retrotransposons were shown be involved in the pathogenicity of *C. albicans* [66] and associated with the rapid evolution of effectors and the adaptation to new host in other pathogenic filamentous fungi [67] and Oomycetes [68]. For insect fungi, it was shown that more than 65 % of the transposase genes were transcribed in *Metarhizium* hyphae during the infection process [2]. Therefore, the enrichment of retrotransposons in *O. polyrhachis-furcata* and *O. sinensis* could be related to their high host specificity and a unique pathogenicity compared to the other insect fungi. The evolution of host specificity and the diversification of insect fungi may thus be driven by the expansion/reduction of these elements.

## Conclusions

The sequencing and annotation of fungal genomes have allowed mycologists to gain insight into the fundamental aspects of fungal and eukaryotic biology. Comparative analyses between *O. polyrhachis-furcata* and some other fungi give unprecedented insights into the biology of this species, particularly regarding the emergence of its extreme host specificity. Gene contractions have been postulated as being one of major causes in the evolution of host-specificity in various organisms. This is due to the fact that losses of genes are accompanied by losses of capacity of the organisms to exploit range of hosts. The contraction of various gene families in *O. polyrhachis-furcata* is in line with this hypothesis. However, this cannot alone explain the evolution of host specificity. Specific genes and even genomic regions or chromosomes are also the origin of host specificity in many fungal pathogens. Some genes found in *O. polyrhachis-furcata* are unique among entomopathogenic fungi. The contribution of these genes to the unique biology and host specificity of *O. unilateralis s.l.* remains to be studied. Particularly, the expansions of genes related to the production of bacterial-like toxins and of retrotransposons are of major interests. While loss of toxins and capacity to kill hosts rapidly was proposed as on mechanism underlying host-specificity [64], the pattern of expansion observed in *O. polyrhachis-furcata* suggest a completely different mechanism. Also, the expansion of retrotransposons in this fungus is unique among insect fungi. These findings will pave a way to a better understanding of this unique organism and provide promising field of research.

## Methods

### Fungal strains

*O. polyrhachis-furcata* (BCC54312) was collected from Khao Yai National Park in Nakhon Ratchasima province of Thailand. The strain was isolated and grown on Grace Insect Cell Medium and PDA according to [69]. After the growth on PDA for two months, the mycelia were harvested separated into several eppendorfs, not to exceed approximately 100 mg per tube. The mycelia were extracted for DNA by DNeasy Plant Mini Kit (QIAGEN) by following the manual for 454 sequencing and also using a modified CTAB extraction for samples aimed for mate-pair sequencing.

### Genome/transcriptome sequencing and assembly

The genomic DNA sample of *Ophicordyceps polyrhachis-furcata* was extracted and used to construct shotgun genomic DNA library according to the GS FLX protocol (Roche). The shotgun reads were *de novo* assembled by Newbler v.2.8. In order to construct scaffolds, the genomic DNA sample was used to construct mate-pair libraries with varied insertion sizes of 3, 6 and 8 kb sequencing which were sequenced on Hiseq2000 (Illumina). The mate-pair reads together with the pre-assembled contigs were then used for scaffolding using SSPACE 2.0 [70].

To obtain the transcriptome, *O. polyrhachis-furcata* (BCC54312) was grown on four different media formulations: PDA, PDA + Bacto Soytone 1 %, PDA + Malt Extract 0.5 % and PDA + Phyto Peptone 2 %. The mycelia from different media were harvested together and frozen with liquid nitrogen until the RNA extraction. Frozen mycelia were grinded and transferred to 2.0 ml tubes. 1 ml of RNA extraction reagent (Invitrogen) was added to each tube, vortexed and added 0.1 ml 5 M NaCl and 0.3 ml Chloroform. The solutions were then centrifuged at 14,000 rpm for 5 min at 4 °C. The supernatants were then transferred to new tubes and added equal volumes of Chloroform (~1 ml), vortexed and centrifuged at 14,000 rpm for 10 min at 4 °C. The chloroform extraction was repeated until there was not interphase anymore. The RNA was precipitated with cold ethanol and 3 M LiCl, incubated at −20 °C overnight. The tubes were then centrifuged at 12,000 rpm for 25 min at 4 °C. The RNA pellets were washed with 70 % ethanol twice and re-suspended in 50 ul DEPC-water.

mRNA (200 ng) was isolated from the total RNA sample using mRNA isolation kit (Stratagene) and subjected to cDNA library construction and sequencing on the GS FLX platform (Roche). We obtained 568,0911 reads with the average read length of 538 bp. The cDNA reads were cleaned (trim poly-A tail, remove short/repetitive/low-quality reads) by SeqClean [http://sourceforge.net/projects/seqclean/] and 535,728 reads were *de novo* assembled into 12,293 contigs by Newbler cDNA *de novo* assembler (Roche). The transcriptome was used to improve the annotation.

### Gene prediction and annotation

MAKER (v.2.28) [71] was used as the main annotation pipeline for gene prediction and annotation. As part of MAKER, GlimmerHMM (v.3.0.1) [72], AUGUSTUS (v.2.6.1) [73, 74], SNAP (released 29/11/2013) [75], and GeneMark-ES fungal version [76] were used as *ab initio* gene predictors. The *ab initio* SNAP was trained for a couple times within MAKER based on sequence similarity search via BLAST [77] and sequence alignment via Exonerate (v.2.2.0) [78] with default parameter settings, using 386,567 EST and 248,253 protein sequences of the order Hypocreales compiled from NCBI in October 2013, together with 10,848 assembled RNA-Seq transcripts, against the 598 scaffolds which were also masked by RepeatMasker (v.4.0.3) [79] together with Tandem Repeat Finder (TRF) (v.4.04) [80] and RMBlast (NCBI blast package of RepeatMasker), based on Repbase database (repeatmaskerlibraries-20130422.tar.gz; [81]). The transposable elements were extracted from the various types of masked repeats. The predicted transcripts were searched against the non-redundant protein database (NR) downloaded from NCBI on Feb 10, 2014. The BLAST results were then imported into BLAST2GO [82, 83] for Gene Ontology (GO), Enzyme, and KEGG pathway annotations. The tRNAs were predicted by the tRNAscan-SE (v.1.3.1) [84] and Aragorn (v.1.2.34) [85] and the protein secretion was predicted by SignalP 4.0 [12]. The completeness of genome assembly and gene annotation was assessed using BUSCO (v.1.1b1) [86], with the 598 scaffolds and predicted protein sequences of *O. polyrhachis-furcata* as respective inputs and the BUSCO’s fungal dataset of 1438 benchmarking universal single-copy orthologs as profile.

### Orthology and phylogenomic analysis

The standalone InParanoid (v.4.1) [38] and QuickParanoid [39] with default parameter settings were respectively used to identify and cluster the orthologous proteins of *O. polyrhachis-furcata* and the nineteen other taxa (the proteomes of these taxa were downloaded from UniProt: Additional file 10: Table S10). The concatenated 1,053 protein sequences conserved among all taxa were used as input of RAxML (v.8) [87] for building the phylogenomic tree using the Dayhoff model.

### Protein family classifications

The potential pathogenic and virulence genes were identified by BLASTP against the pathogen-host interaction database (PHI-base) v.3.5 [13]. The families of proteases were identified by BLASTP against the peptidase database (MEROPS) release 9.10 [88, 89] with the E-value cutoff < = 1E-20. The carbohydrate active enzymes; glycoside hydrolases (GHs), polysaccharide lyases (PLs), carbohydrate esterases (CEs), were classified by the CAZy database [90], of which protein sequences were manually compiled via a CAZy tool [91] kindly provided by Alexander Holm Viborg. The G-protein coupled receptors and transporters were classified based on BLASTP against the GPCRDB [92] (version 2013.09.26) and Transporter Classification Database (TCDB) (Last modified: July 15, 2011) [93], respectively. BLASTP against all specific protein databases except MEROPS used E-value cutoff < = 1E-5. Gene clusters and core genes for biosynthesis of secondary metabolites were identified by SMURF [55] and antiSMASH [94]. For phylogenetic analysis, sequences of all species hit with specific protein family (i.e., MEROPS: S08 and S53 for subtilisins) were extracted and multiple aligned using MAFFT v.7 [95]. RAxML version 8 [87] was then used to analyze the phylogenetic trees based on maximum likelihood and visualized by Dendroscope 3 [96].

## Additional files

Additional file 1: Table S1. The number of genes for PHI genes family identified in *O. polyrhachis-furcata* and nineteen other fungi. (XLSX 252 kb) Additional file 2: Table S2. The blast results for the protein-coding genes of *O. polyrhachis-furcata* against PHI database. (XLS 385 kb) Additional file 3: Table S3. The number of subtilisins, trypsins, and aspartyl proteases identified in *O. polyrhachis-furcata* compared with nineteen other fungi. (XLSX 14 kb) Additional file 4: Table S4. The number of genes following the MEROPS databases (peptidases) identified in *O. polyrhachis-furcata*, compared with the nineteen other fungi included in this study. (XLSX 50 kb) Additional file 5: Table S5. The number of genes in different Glycoside Hydrolase (GH) families, following the Carbohydrate-Active Enzymes Databases (CAZY), identified in *O. polyrhachis-furcata* and other nineteen fungi included in this study. (XLSX 21 kb) Additional file 6: Table S6. The number of genes in different Polysaccharide Lyase (PL) families, following the Carbohydrate-Active Enzymes Databases (CAZY), identified in *O. polyrhachis-furcata* and other nineteen fungi included in this study. (XLSX 10 kb) Additional file 7: Table S7. The number of genes in different Carbohydrate Esterase (CE) families, following the Carbohydrate-Active Enzymes Databases (CAZY), identified in *O. polyrhachis-furcata* and other nineteen fungi included in this study. (XLSX 10 kb) Additional file 8: Table S8. The number of bacterial-like toxins identified in *O. polyrhachis-furcata* compared with other entomopathogenic fungi. (XLSX 15 kb) Additional file 9: Table S9. The number of transposable elements (TEs) identified in OPF compared with other entomopathogenic fungi. The data of *B. bassiana*, *C. militaris*, *M. robertsii*, and *M. acridum* were obtained from Supplemental Table S2 of [3]. The data of *M. anisopliae* was obtained from [2]. The data of *O. sinensis* was obtained from [4]. (PDF 10 kb) Additional file 10: Table S10. List of nineteen fungal species selected from UniProt for orthology and comparative analysis with *O. polyrhachis-furcata* BCC54312. (PDF 29 kb)
