# Supplementary material for: Insights from the genome of Ophiocordyceps polyrhachis-furcata to pathogenicity and host specificity in insect fungi
Source: BMC Genomics. 2015 Oct 28;16:881. doi: 10.1186/s12864-015-2101-4 (PMC4625970; doi:10.1186/s12864-015-2101-4)
Supplement: Additional file 9: Table S9. — The number of transposable elements (TEs) identified in OPF compared with other entomopathogenic fungi. The data of B. bassiana, C. militaris, M. robertsii, and M. acridum were obtained from Supplemental Table S2 of [3]. The data of M. anisopliae was obtained from [2]. The data of O. sinensis was obtained from [4]. (PDF 10 kb) [file 12864_2015_2101_MOESM9_ESM.pdf]

Table S9. The number of transposable elements (TEs) identified in *O. polyrhachis-furcata* compared with other entomopathogenic fungi.

Classes	*B. bassiana*	*C. militaris*	*M. robertsii*	*M. acridum*	*O. sinensis*	*O. polyrhachis-furcata*
DNA/hAT	37	2	45	2	2	23
DNA/Mariner	10	1	15	4	247	7
DNA/MuDR	5	1	14	1	276	11
DNA/Helitron	0	0	21	5	2	7
LTR/Copia	3	0	19	0	8353	77
LTR/Gypsy	4	0	6	5	15357	101
NonLTR/LINE	29	0	28	3	1556	65
