# Supplementary material for: Insights from the genome of Ophiocordyceps polyrhachis-furcata to pathogenicity and host specificity in insect fungi
Source: BMC Genomics. 2015 Oct 28;16:881. doi: 10.1186/s12864-015-2101-4 (PMC4625970; doi:10.1186/s12864-015-2101-4)
Supplement: Additional file 10: Table S10. — List of nineteen fungal species selected from UniProt for orthology and comparative analysis with O. polyrhachis-furcata BCC54312. (PDF 29 kb) [file 12864_2015_2101_MOESM10_ESM.pdf]

Table S10. List of fungal species included in this study

Full scientific name	Abbreviated name	Full name at UniProt
Aspergillus fumigatus	ASF	Neosartorya fumigata (strain ATCC MYA-4609 / Af293 / CBS 101355 / FGSC A1100) (Aspergillus fumigatus)
Aspergillus nudulans	ASNIA	Emericella nidulans (strain FGSC A4 / ATCC 38163 / CBS 112.46 / NRRL 194 / M139) (Aspergillus nidulans)
Batrachochytrium dendrobatidis	BATD	Batrachochytrium dendrobatidis (strain JAM81 / FGSC 10211) (Frog chytrid fungus)
Beauveria bassiana	BEUBAS	beauveria bassiana (strain arsef 2860) (white muscardine disease fungus) (tritirachium shiotae)
Botryotinia fuckeliana	BOTFW	Botryotinia fuckeliana (strain BcDW1) (Noble rot fungus) (Botrytis cinerea)
Candida albicans	CANAlB	Candida albicans (strain SC5314 / ATCC MYA-2876) (Yeast)
Coprinopsis cinerea	COPCIN	Coprinopsis cinerea (strain Okayama-7 / 130 / ATCC MYA-4618 / FGSC 9003) (Inky cap fungus) (Hormographiella aspergillata)
Cordyceps militaris	CORMIL	Cordyceps militaris (strain CM01) (Caterpillar fungus)
Fusarium graminearum	FUSGRA	Gibberella zeae (strain PH-1 / ATCC MYA-4620 / FGSC 9075 / NRRL 31084) (Wheat head blight fungus) (Fusarium graminearum)
Magnaporthe oryzae	MAGORY	Magnaporthe oryzae (strain 70-15 / ATCC MYA-4617 / FGSC 8958) (Rice blast fungus) (Pyricularia oryzae)
Metarhizium acridum	METACR	Metarhizium acridum (strain CQMa 102)
Metarhizium robertsii	METARB	Metarhizium robertsii (strain ARSEF 23 / ATCC MYA-3075) (Metarhizium anisopliae (strain ARSEF 23))
Neurospora crassa	NEUCRA	Neurospora crassa (strain ATCC 24698 / 74-OR23-1A / CBS 708.71 / DSM 1257 / FGSC 987)
Ophiocoryceps polyrhachis-furcata	OPF	-
Ophiocordyceps sinensis	OPSINE	Ophiocordyceps sinensis (strain Co18 / CGMCC 3.14243) (Yarsagumba caterpillar fungus) (Hirsutella sinensis)
Saccharomyces cerevisiae	SACCES	Saccharomyces cerevisiae (strain ATCC 204508 / S288c) (Baker's yeast)
Schizosaccharomyces pombe	SCHIPO	Schizosaccharomyces pombe (strain 972 / ATCC 24843) (Fission yeast)
Sclerotinia sclerotiorum	SCLSCL	Sclerotinia sclerotiorum (strain ATCC 18683 / 1980 / Ss-1) (White mold) (Whetzelinia sclerotiorum)
Ustilago maydis	USTMAY	Ustilago maydis (strain 521 / FGSC 9021) (Corn smut fungus)
Verticillium alfalfae	VERALB	Verticillium alfalfae (strain VaMs.102 / ATCC MYA-4576 / FGSC 10136) (Verticillium wilt of alfalfa) (Verticillium albo-atrum)
